# Nicotine Deteriorates the Osteogenic Differentiation of Periodontal Ligament Stem Cells through α7 Nicotinic Acetylcholine Receptor Regulating wnt Pathway

**DOI:** 10.1371/journal.pone.0083102

**Published:** 2013-12-20

**Authors:** Zhifei Zhou, Bei Li, Zhiwei Dong, Fen Liu, Yu Zhang, Yang Yu, Fengqing Shang, Lizheng Wu, Xiaojing Wang, Yan Jin

**Affiliations:** 1 Department of Pediatric Dentistry, School of Stomatology, Fourth Military Medical University, Xi’an, Shaanxi, China; 2 Research and Development Center for Tissue Engineering, Fourth Military Medical University, Xi’an, Shaanxi, China; 3 Department of Oral Histology and Pathology, School of Stomatology, Fourth Military Medical University, Xi’an, Shaanxi, China; 4 Department of Oral and Maxillofacial Surgery, General Hospital of Shenyang Military Area Command, Shenyang, Liaoning, China; 5 Department of Oral Medicine, Maternal and Child Care Hospital, Xi’an, Shaanxi, China; 6 Department of Orthodontic, School of Stomatology, Fourth Military Medical University, Xi’an, Shaanxi, China; University of Massachusetts Medical, United States of America

## Abstract

**Aims:**

Cigarette smoking is one of the high risk factors of adult chronic periodontitis and nicotine is the well established toxic substance in cigarette. However, the mechanism of nicotine induced periodontitis is still unknown. Here we studied whether nicotine impaired the osteogenic differentiation of human periodontal ligament stem cells (hPDLSCs) through activating α7 nicotinic acetylcholine receptor (α7 nAChR).

**Methods:**

hPDLSCs with multi differentiation potential and surface makers for mesenchymal stem cells were harvested by limiting dilution technique. The level of mineralized nodule formation was assessed by alizarin red S staining. Expression level of ostegenic related genes and proteins were detected by real-time PCR and western blot analysis. The expression of α7 nAChR and its downstream signaling pathway were examined by western blot. The role of the receptor and related signaling pathway in nicotine impairing the osteogenic potential of hPDLSCs were also studied in different levels.

**Results:**

Nicotine deteriorated the ostegenic differentiation of hPDLSCs in a dose dependent manner. Activation of α7 nAChR by nicotine treatment activated wnt/β-catenin signaling pathway, leading to osteogenic deficiency of hPDLSCs. Blockage of α7 nAChR and wnt pathway inhibitor treatment rescued nicotine induced osteogenic differentiation deficiency.

**Conclusions:**

These data suggested that nicotine activated α7 nAChR expressed on PDLSCs and further activated wnt signaling downstream, thus deteriorating the osteogenic potential of PDLSCs. The impairment of osteogenic differentiation of PDLSCs by nicotine might lead to cigarette smoking related periodontitis.

## Introduction

Periodontitis is a chronic disease which results in the loss of periodontal tissues in clinical symptoms of periodontal attachment loss, forming of periodontal pocket, osteopenia for alveolar bone and finally teeth exfoliation [1]. Periodontal ligament stem cells (PDLSCs), a newly recognized subpopulation of mesenchymal stem cells (MSCs), have been isolated from periodontal ligament tissues and are capable of regenerating cementum/periodontal ligament tissues *in vivo* [2]. Further studies confirmed that the complex formed by hPDLSCs was similar to natural periodontal tissues in considering histological morphology and spatial arrangement [3]. All these results indicated that PDLSCs had the characteristic of differentiating into osteoblast like cells which was critical to the regeneration of periodontal tissues.

Cigarette smoking has been recognized as a high risk factor for periodontitis [4]. Not only could it lead to a high prevalence of periodontal diseases, but also smoking would deteriorate the prognosis of periodontitis even if comprehensive treatments were received [5]. Potential mechanisms for this phenomenon could be considered as that nicotine could stimulate periodontal ligament cells to express inflammatory factors such as IL-8 thus destructing the stable status of periodontal tissues [6] and the balance of immunoregulation in local microenvironment [7]. In addition, it is recognized that besides bone absorption, nicotine also down regulated the volume of newly formed alveolar bone tissues [8].

Our previous research suggested that α7 nicotinic acetylcholine receptor (α7 nAChR), which was an important receptor for nicotine, expressed in periodontal tissues in rats and human beings [9] after study confirmed that nAChR expressed in non neuron tissues [10]. We also found that the adverse effects of nicotine on periodontal tissues could be antagonized by α-Bungarotoxin (α-BTX), a specific antagonist of α7 nAChR. Thus the signaling pathway of α7 nAChR might be one of the potential mechanisms for impaired bone formation of nicotine related periodontitis.

However, the mechanism of smoking related periodontitis is still unclear. Previous study has reported that nicotine up regulated wnt signaling pathway in human alveolar interstitial fibroblast [11]. And our previous results have already demonstrated that the activation of wnt signaling pathway suppressed the osteogenic potential of PDLSCs under both normal and inflammatory circumstances [12]–[14].

Thus hypotheses were made that the specific receptor of nicotine α7 nAChR expressed in human periodontal ligament stem cells through which nicotine affected wnt signaling pathway downstream and further regulated the osteogenic capacity of PDLSCs. We reported that nicotine deteriorated osteogenic differentiation of hPDLSCs in a dose dependent manner. The impairment of ostegenic differentiation of hPDLSCs was mediated through activated α7 nAChR and downstream wnt/β-catenin pathway.

## Materials and Methods

### Study Subjects and Ethics Statement

Normal healthy premolars and third molars (n = 14) were obtained from 8 patients at the age of 12 to 23 years old who needed to extract the teeth because of orthodontic purposes and 9 teeth of them were used for further experiment. All the subjects were from School of Stomatology, Fourth Military Medical University and were free from any recent clinical acute or chronic infections. Before the investigation, the participants and guardians on behalf of them were informed of the objectives of this study. Written consents were obtained from them prior to conducting the study. Ethical approval had been obtained from the Ethics Committee of School of Stomatology, Fourth Military Medical University.

### Cell Culture

PDLSCs were isolated and cultured as we previously described [15]–[18]. All the teeth were washed by sterile phosphate buffer solution (PBS) and then periodontal tissues from the middle third of the roots were gently scratched and cut into small pieces (1 mm^3^). Gingival or dental pulp tissues were excluded. Scratched tissues were digested with Type I Collagenase (Sigma, Santa Clara, CA, USA) for 15 minutes, then re-suspended in Alpha Minimum Essential Medium (α-MEM, Hyclone, Logan, Utah, USA) which containing 10% (v/v) fetal bovine serum (FBS, Hyclone, Logan, Utah, USA), 0.292 mg/mL glutamine (Invitrogen, Carlsbad, CA, USA), 100 U/mL penicillin (Invitrogen, Carlsbad, CA, USA) and 100 mg/mL streptomycin (Invitrogen, Carlsbad, CA, USA). Periodontal ligament tissues were planted in 6-well plate at 37°C in a humidified atmosphere of 95% air and 5% CO_2_. Medium was changed every 3 days. When cells surrounding the explants reached confluence, cell layers were harvested by 0.25% pancreatin (pH = 8.0–9.0, Sigma, Santa Clara, CA, USA) taking the technique of limiting dilution to pick up monoplast colony for further culturing of hPDLSCs *in vitro*. Single-cell derived colonies were used in this study after 2–4 passages. For each experiment, PDLSCs in the same passage were used.

### Immunophenotype Analysis

PDLSCs were stained with antibodies for stem cell surface markers and analyzed by flow cytometry as described previously [19]–[21]. Briefly, To identify the phenotypes of hPDLSCs, 5×10^5^ cells at the 3^rd^ passage were incubated with phycoerythrin (PE) conjugated monoclonal antibodies for human CD146 (Biolegend, San Diego, CA, USA), CD34 (Biolegend, San Diego, CA, USA), CD31 (eBioscience, San Diego, CA, USA) and fluorescein isothiocynante (FITC) conjugated monoclonal antibodies for human stro-1 (Biolegend, San Diego, CA, USA), CD105 (eBioscience, San Diego, CA, USA), CD90 (eBioscience, San Diego, CA, USA), CD44 (Abcam, Cambridge, UK), CD14 (eBioscience, San Diego, CA, USA) as the manufacturer’s instructions. The incubation procedure was carried out at 4°C away from light for 1 hour. After washing with PBS, cells were subjected to flow cytometric analysis (Beckman Coulter, Fullerton, CA, USA).

### Osteogenic/Adipogenic Differentiation of hPDLSCs

Osteogenic/Adiopogenic differentiations of PDLSCs were performed according to previous publications [2], [16], [22]. Briefly, 5×10^4^ cells per well were seeded to 6-well plates and cultured in α-MEM containing 10% FBS, 0.292 mg/mL glutamine, 100 U/mL penicillin, 100 mg/mL streptomycin. Osteogenic medium containing 100 nm dexamethasone (Sigma, Santa Clara, CA, USA), 50 ug/mL ascorbic acid (Sigma, Santa Clara, CA, USA) and 5 mM β-glycerophosphate (Sigma, Santa Clara, CA, USA) or adipogenic medium containing 0.5 mM methylisobutylxanthine (Sigma, Santa Clara, CA, USA), 0.5 mM hydrocortisone (Sigma, Santa Clara, CA, USA), 60 mM indomethcin (Sigma, Santa Clara, CA, USA) was given when cells reached confluence and the conditioned medium was changed every 3 days. After 21 days of conditioned culturing, cells were fixed with 75% ethanol and stained with oil red O solution and 2% alizarin red (Sigma, Santa Clara, CA, USA) respectively. After washing with PBS, the cells were observed using an inverted microscope and imaged. The nodule area per well were measured quantitatively with an image analysis system Image-Pro Plus 5.0 software.

### WST-1 Cell Proliferation Assay

The toxic effect of nicotine (Sigma, Santa Clara, CA, USA) on hPDLSCs was detected with a modified MTT assay using WST-1 cell cytotoxicity assay kit (Roche Applied Science, Mannheim, Germany). 5×10^3^ cells per well were cultured in 96-well plates in 100 uL medium. After 24 hours cells were administrated with nicotine at different concentrations. 10 uL WST-1 was added to each well at the same time point everyday for 7 days and incubated for another 4 hours away from light in humidified atmosphere. After gentle vibrating for 1 minute, microtiter plate reader (Bio-Rad Model 680, Life Science Research, Tokyo, Japan) was used to measure the sample under wavelength of 450 nm with a 620 nm reference wavelength.

### Total RNA Extraction and Quantitative Real Time Polymerase Chain Reaction

Total RNA of hPDLSCs was isolated using Trizol reagent (Invitrogen, Carlsbad, CA, USA). Approximately 3 ug total RNA of each experimental group was converted to cDNA with the Super Script First Strand Synthesis Kit (TaKaRa, Dalian, China). Real time polymerase chain reaction (PCR) system was prepared using QuantiTect SYBR Green PCR kit (TaKaRa, Dalian, China) and applied Bio-systems CFX96™ Real-Time sequence detection system (Applied Biosystems, Darmstadt, Germany). Primers used in this study were listed in Table 1. For each detected gene, three independent experiments were carried out with different groups of hPDLSCs.

**Table 1:** Primer sequences.

Gene	Primers
h-ALP-F	CCTTGTAGCCAGGCCCATTG
h-ALP-R	GGACCATTCCCACGTCTTCAC
h-Ocn-F	CCCAGGCGCTACCTGTATCAA
h-Ocn-R	GGTCAGCCAACTCGTCACAGTC
h-Runx2-F	CACTGGCGCTGCAACAAGA
h-Runx2-R	CATTCCGGAGCTCAGCAGAATAA
h-BSP-F	GGGCAGTAGTGACTCATCCGA
h-BSP-R	TCTTCATTGTTTTCTCCTTCATTTG
h-β-actin-F	TGGCACCCAGCACAATGAA
h-β-actin-R	CTAAGTCATAGTCCGCCTAGAAGCA

### Protein Isolation and Western Blot Analysis

Total protein was isolated from hPDLSCs using lysis in RIPA buffer (10 mM Tris-HCL, 1 mM EDTA, 1% sodium dodecyl sulfate, 1% Nonidet P-40, 1∶100 proteinase inhibitor cocktail, 50 mM β-glycerophosphate, 50 mM sodium fluoride) on ice. The lysate was incubated for 20 minutes and then centrifuged at 12000 rpm for 15 minutes at 4°C. Protein quantification was performed by using a protein assay solution (Beyotime, Suzhou, China) to detect the absorbance at 595 nm wavelength. Aliquots of 40 ug protein per sample was separated by 10% sodium dodecyl sulfate-polyacrylamide gel electrophoresis (SDS-PAGE) and then transferred onto a polyvinylidene fluoride (PVDF) membrane (Millipore, Temecula, CA, USA). The membrane was blocked in 5% (w/v) milk protein dissolved in PBS for 2 hours and then incubated with primary antibody overnight at 4°C. Subsequently, the sample was washed in Tween-TRIS buffered saline (TTBS, 10 mM TRIS-HCl, 50 Mm NaCl, 0.25% Tween) and incubated with horseradish peroxidase-conjugated secondary antibody for 1 hour at room temperature. Immunodetection was performed using the Western-Light Chemiluminescent Detection System to detect the specific protein band on the membrane. Primary antibodies were purchased from the following commercial sources: antibodies against ALP, OCN and DKK1 were from Abcam, Cambridge, UK; antibodies against BSP, Runx2, α7 nAChR and p-GSK-3β were from santa cruz, Dallas, Texas, USA; antibodies against β-catenin and GSK-3β were from cell signaling, Danvers, MA, USA; antibodies against active-β-catenin was from Millipore, Temecula, CA, USA.

### Nicotine, α-BTX and DKK1 Treatment

Nicotine at the concentrations of 10^−3^, 10^−4^, 10^−5^, 10^−6^, 10^−7^ mol/L was administrated to the culturing medium of hPDLSCs when they were attached. The morphology of cells was observed and the proliferation of hPDLSCs was examined after 24 hours. In further study for osteogenic differentiation, 5×10^4^ cells/well were seeded to a 6-well plate and cultured in α-MEM. When cells reached confluence, the conditioned osteogenic medium was changed with nicotine at the concentrations of 10^−4^, 10^−5^, 10^−6^ mol/L. 10^−8^ mol/L α-BTX (Toctis, Bristol, UK) or 10 ng/mL DKK1 (Pepro Tech, Suzhou, China) was added together with nicotine.

### Statistical Analysis

All experiments were performed in triplicate with three different groups of hPDLSCs. Each data was expressed in a mean ± standard deviation (SD) form. The statistical differences between two groups were determined using two-tailed unpaired Students’s t test while that for more than two groups one way Analysis of Variance (ANOVA) was used followed by turkey post-test. SPSS 13.0 software was utilized and p value less than 0.05 were considered as statistically significant.

## Results

### Nicotine Suppressed the Proliferation of hPDLSCs in a Dose Dependent Manner

**Figure 1:**
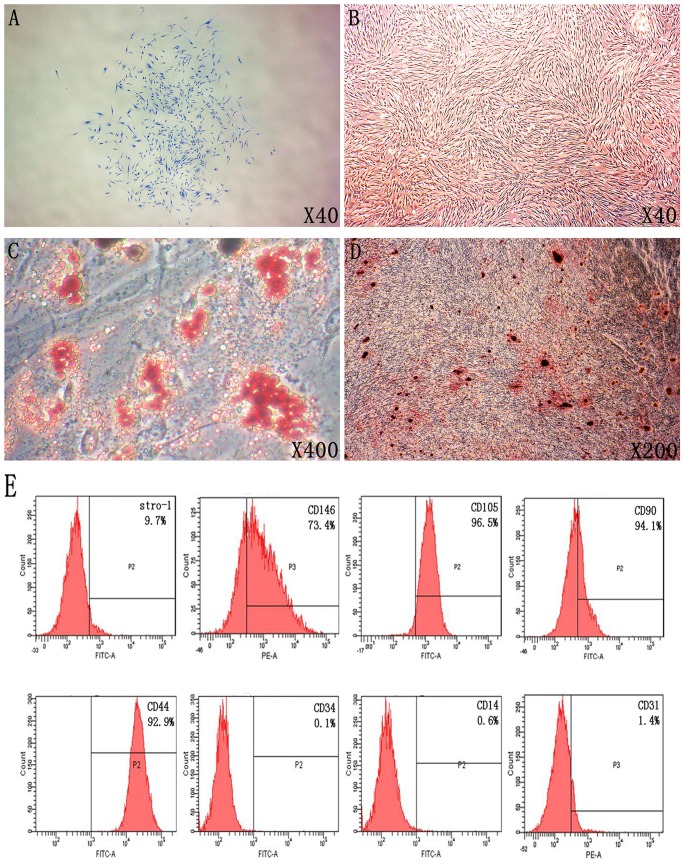
Isolation and identification for human periodontal ligament stem cells. **A**: Colonies derived from single cell in the method of limiting dilution was picked up for further culturing of hPDLSCs; **B**: hPDLSCs were grown in culture medium with a long spindle shape; **C**: Adipogenic differentiation of hPDLSCs was determined by Oil red O staining after 21 days of adipogenic induction; **D**: Osteogenic differentiation of hPDLSCs was determined by Alizarin red S staining after 21 days of osteogenic induction; **E**: Immunophenotype analysis of PDLSCs was determined by Flow cytometry assay.

First human PDLSCs which could form single cell colony (Fig. 1A) and express mesenchymal stem cell markers including CD44, CD90, CD105, CD146 and Stro-1; but were negative for hematopoietic cell marker CD34, monocytes maker CD14 and platelet endothelial cell maker CD31 (Fig. 1E) were isolated and characterized. Under the microscope hPDLSCs were grown in an adherent way with a long spindle shape and a radial arrangement (Fig. 1B). These cells showed osteogenic and adipogenic differentiation capacity after induction for 3 weeks *in vitro* (Fig. 1C, D). The results also showed no difference of osteogenic differentiation of PDLSC from 9 different donors (Figure S1).

**Figure 2:**
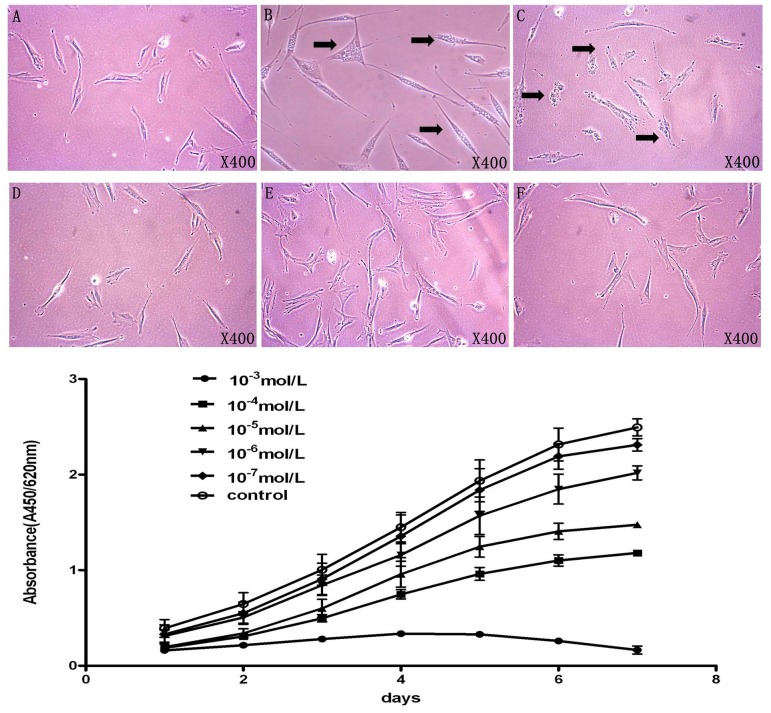
Nicotine affects the morphology and suppresses the proliferation of hPDLSCs. **A**: Morphology of PDLSC in control group; **B**: morphology of hPDLSC with stimulation of nicotine in 10^−3^ mol/L, black arrow indicated the vacuolar degeneration of PDLSCs; **C–F**: Morphologies of hPDLSC with stimulation of nicotine from 10^−4^–10^−7^ mol/L, increased granular substance in PDLSCs was indicated by black arrow; **G**: Proliferation of hPDLSCs from each group was determined by WST-1 assay. Nicotine with different concentrations suppresses the proliferation of hPDLSC.

After 24 hours seeded in a 6-well plate, hPDLSCs could be observed in long spindle morphology with clear cell nucleus (Figure 2A). However when 10^−3^ mol/L nicotine was added into the α-MEM medium in 24 hours, vacuolar degeneration could be observed (Figure 2B). Meanwhile when the concentration of nicotine was 10^−4^ mol/L, increased granular substances could be clearly found in cell cytoplasm (Figure 2C). There is no significant difference in cell morphology comparing 10^−5^, 10^−6^, 10^−7^ mol/L nicotine treatment groups to the control group (Figure 2D–F).

In addition, the proliferation of hPDLSCs was examined after treated by 10^−3^, 10^−4^, 10^−5^, 10^−6^, 10^−7^ mol/L nicotine respectively. WST-1 cell proliferation assay showed that when 10^−3^ mol/L nicotine was added into the medium, the proliferation rate of hPDLSCs decreased significantly compared to the control group (Fig. 2G). 10^−4^ to 10^−6^ mol/L nicotine treatment also decreased the proliferation of hPDLSCs while 10^−7^ mol/L nicotine didn’t affect the cell proliferation (Fig. 2G). Thus, nicotine suppressed the proliferation of hPDLSCs in a dose dependent manner.

### Nicotine Deteriorated the Osteogenic Differentiation Capacity of hPDLSCs

**Figure 3:**
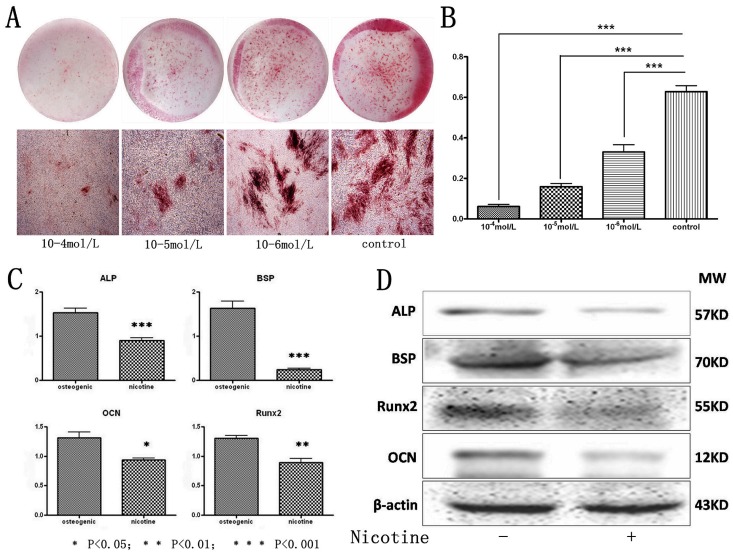
Nicotine deteriorates the osteogenic differentiation capacity of hPDLSCs. **A**: hPDLSCs treated by 10^−4^–10^−6^ mol/L nicotine were cultured in osteogenic medium and osteogenic differentiation was determined by Alizarin red S staining after 21 days. Representative entire plate views of alizarin red staining in 6-well plates for PDLSCs from each group. **B**: The quantity result for Alizarin Red S staining; **C**: The expression of osteogenic related genes Runx2, BSP, ALP and OCN were determined by qRT-PCR after 14 days of culturing with osteogenic supplement. **D**: The expression of osteogenic related protein Runx2, BSP, ALP and OCN were determined by western blot after 14 days of culturing with osteogenic supplement. Data represent the means ± SD. *p<0.05 (n = 3).

To identify the role of nicotine in regulating the osteogenic differentiation capability of hPDLSCs, different doses of nicotine were added to the osteogenic induction medium. Alizarin Red S staining showed that nicotine impaired osteogenic differentiation ability for hPDLSCs in a dose dependent manner while 10^−4^ mol/L nicotine inhibited hPDLSCs to form mineralized nodules most significantly after 3 weeks’ induction (Fig. 3A, B). In addition, hPDLSCs in different groups were also stained for qualitative ALP staining after 7 days of osteogenic induction; the results were identical with Alizarin Red S staining (Figure S2).

Also in the concentration of 10^−4^ mol/L, nicotine could down regulate the expression of osteogenic related genes like ALP, OCN, BSP and Runx2 compared with the control group with a statistical significance (p<0.05, Figure 3C). In the same concentration of nicotine, the protein expression of ALP, OCN, BSP and Runx2 during the osteogenic induction also decreased (P<0.05, Figure 3D).

### Nicotine Impaired Osteogenic Differentiation of hPDLSCs through the Activation of α7 nAChR

**Figure 4:**
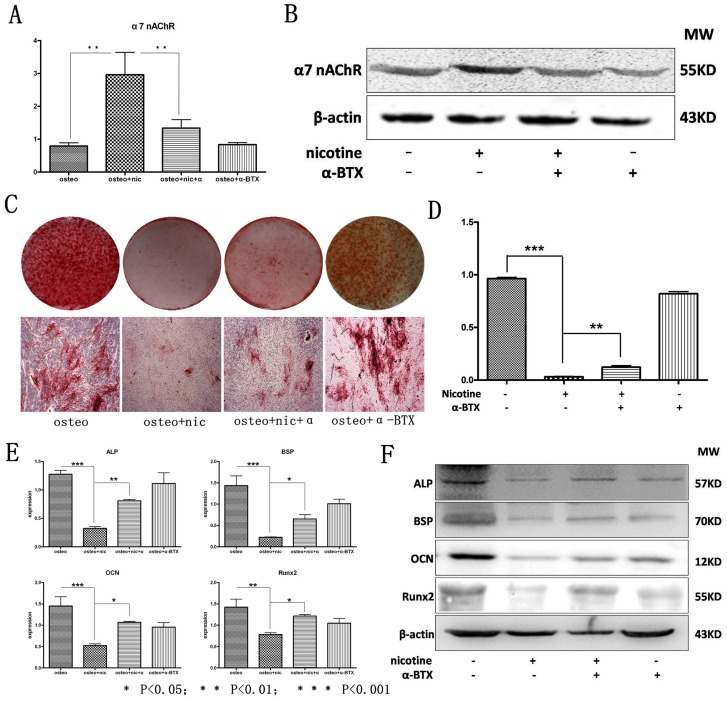
Nicotine deteriorates the osteogenic differentiation of hPDLSCs through the activation of α7 nAChR. The expression of α7 nAChR in PDLSCs from each group was detected by (**A**) qRT-PCR and (**B**) Western Blot analysis. **C, D**: hPDLSCs treated with/without nicotine and α-BTX were cultured in osteogenic medium and osteogenic differentiation was determined by Alizarin red S staining after 21 days. **E**: The expression of osteogenic related genes Runx2, BSP, ALP and OCN were determined by qRT-PCR after 14 days of culturing with osteogenic supplement. **F**: The expression of osteogenic related protein Runx2, BSP, ALP and OCN were determined by western blot after 14 days of culturing with osteogenic supplement. Data represent the means ± SD. *p<0.05 (n = 3).

Our previous research indicated that α7 nAChR expressed in human periodontal ligament fibroblasts and rat periodontal tissues [9]. Here we examined whether the deterioration of osteogenic differentiation of hPDLSCs by nicotine was through the activation of α7 nAChR. Results indicated that α7 nAChR expressed in hPDLSCs by qPCR and Western blot analysis (Figure 4A, B). Moreover, its expression was further strengthened with its agonist nicotine (10^−4^ mol/L) while the elevated expression was alleviated when given the specific antagonist of the receptor α-BTX (10^−8^ mol/L) (Figure 4A, B). Meanwhile, there was no statistical significant difference when compared the control group for the expression of α7 nAChR with the group of antagonist α-BTX alone.

Osteogenic induction was carried out within the same groups. After 21 days of osteogenic induction, Alizarin Red S staining was performed and results showed that nicotine significantly inhibited the ability of hPDLSCs to form mineralized nodules while this down regulating effect could be partially reversed by α-BTX. α-BTX alone, however, did not up regulate the capability of hPDLSCs to form mineralized nodules (Figure 4C, D). After 7 days, the results of qualitative ALP staining also indicated that compared with the normal control, nicotine significantly inhibited the ability of hPDLSCs to form ALP in the early period of osteoblast while this down regulating effect could be antagonized by α-BTX which was in accordance with the results of Alizarin Red S staining (Figure S2).

Results of qPCR indicated the same results with histological staining (Figure 4E). Compared with normal control, 10^−4^ mol/L nicotine significantly suppressed the gene expression in osteogenic differentiation such as ALP, OCN, BSP and Runx2. And if given α-BTX (10^−8^ mol/L), the suppressive effect of nicotine was alleviated while α-BTX alone could not up regulate these genes’ expression in osteogenic induction. Results in the protein level were similar to qPCR between the same groups, protein expression of ALP, OCN, BSP and Runx2 showed the same tendency (Figure 4F).

### Nicotine Deteriorated the Osteogenic Differentiation of hPDLSCs through α7 nAChR Regulating wnt/β-catenin Pathway

**Figure 5:**
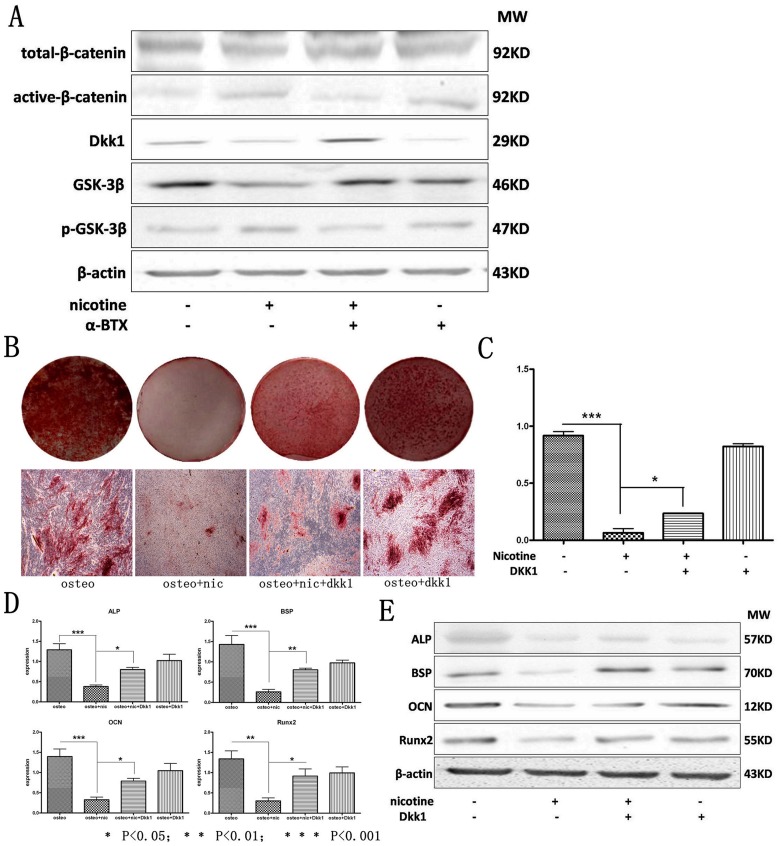
Nicotine deteriorates the osteogenic differentiation of hPDLSCs through α7 nAChR regulating Wnt/β-catenin pathway. **A**: The expression of wnt signaling pathway related proteins were determined by western blot, wnt signaling pathway was activated by nicotine in the downstream of α7 nAChR; **B, C**: hPDLSCs treated with/without nicotine and dkk1 were cultured in osteogenic medium and osteogenic differentiation was determined by Alizarin red S staining after 21 days. **D**: The expression of osteogenic related genes Runx2, BSP, ALP and OCN were determined by qRT-PCR after 14 days of culturing with osteogenic supplement. **E**: The expression of osteogenic related proteins Runx2, BSP, ALP and OCN were determined by western blot after 14 days of culturing with osteogenic supplement. Data represent the means ± SD. *p<0.05 (n = 3).

Our previous study indicated that the activation of wnt/β-catenin pathway suppressed osteogenic differentiation of hPDLSCs [12]. Thus we examined if wnt/β-catenin pathway was downstream regulated by α7 nAChR in hPDLSCs. The protein expression of active-β-catenin was increased under the treatment of nicotine while the effect could be antagonized if co-treated with α-BTX. α-BTX alone didn’t decrease the protein expression of active-β-catenin in hPDLSCs (Figure 5A). In this study, the protein expression of total-β-catenin had no statistical significant difference within the four groups (Figure 5A). The expression of dkk1, which was a suppressive protein of wnt signaling pathway, decreased after treatment of nicotine compared with normal control. While this suppressive effect was reversed when co-treated by α-BTX (Figure 5A). In cytoplasm, β-catenin was continually degraded by a series complexes including GSK-3β, so that the wnt signaling pathway remained inactivated [23]. As the protein expression results indicated, GSK-3β which was included in degradation complexes was down regulated by treatment of nicotine while α-BTX reversed this effect. However α-BTX alone didn’t enhance the protein expression of GSK-3β (Figure 5A). Results of western blot also showed that the expression of phosphorylated-GSK-3β in hPDLSCs was increased after treatment of nicotine while α-BTX co-treatment decreased the expression of phosphorylated-GSK-3β (Figure 5A).

Results of Alizarin Red S staining (Figure 5B, C) revealed that the down regulating effect of nicotine toward the osteogenic differentiation of hPDLSCs was reversed when treated by dkk1 while dkk1 treatment alone had no effect in up regulating the osteogenic differentiation of hPDLSCs. Expression of osteogenic genes and proteins were in accordance with the histological staining. Nicotine suppressed the expression of ALP, OCN, BSP and Runx2 in gene and protein levels while this effect was partially reversed when dkk1 was added simultaneously with nicotine in the osteogenic induction medium. Results also indicated that dkk1 alone didn’t enhance the expression of these genes and proteins (Figure 5D, E).

## Discussion

Every year, the number of people who die from cigarette smoking is about 5 million. It is no doubt that smoking is a risk factor for series systematic diseases such as tumor, respiratory diseases and cardiovascular diseases [24]. For periodontitis, smoking affects its development noted as more severe alveolar bone resorption, loss of periodontal attachment and deeper periodontal pockets [25]. Results of epidemiological investigations which were based on cross sectional studies indicated that prevalence of periodontitis for smokers was 2–7 times more than non-smokers [26] and cigarette smoking played a negative role in teeth preserving for periodontitis patients [27], [28]. Previous studies had already demonstrated that nicotine in cigarette could deteriorate the structure of periodontal fibroblast and further impair the normal function of cells, thus making fibroblast loss their attachment to the root surface and finally leading to a worse recovery for periodontal tissues [9]. Not only the direct effect of inducing cell apoptosis, nicotine could also stimulate neutrophile granulocyte to express inflammatory related cytokine IL-8 in a time and dose dependent manner [29]. Makino et al [30] injected nicotine into abdominal subcutaneous of rats and used enzyme-linked immunosorbent assay to study the development of periodontitis in protein level. Results indicated higher expression of IL-6, IL-10 and IFN-γ in periodontal tissues. However, studies at present mainly focus on the destructive effects of nicotine while few articles cover the field of detailed mechanism for nicotine’s suppressive effect toward the regeneration of periodontal tissues. In current studies, we confirmed that nicotine with different concentrations could suppress the osteogenic potential of PDLSC in a concentration dependent characteristic. Nicotine suppressed PDLSC osteogenic differentiation through the activation of α7 nAChR which might increase phosphoralation of GSK3β and lead to activation of wnt/β-catenin signaling pathway.

PDLSCs possess the basic characteristics of normal stem cells. *In vivo* study had already demonstrated that PDLSCs had the capability of forming cementoblast like cells and cementum/periodontal ligament like tissues [2]. Similar conclusions were also confirmed in mini-swine [31]. The other experiment affirmed that PDLSCs from mini-swine could be autografted and form root/periodontal ligament like complexes which could support artificial tooth root to perform like a natural one [32]. Besides the periodontal tissue regenerative potential for PDLSCs, they could also be used in the field of tooth regeneration by co-culturing with some other stem cells and precursor cells coated outside scaffold materials [33]. Results of Ma et al [34] revealed that PDLSCs co-cultured with cementoblast could accelerate their differentiation procedure in the environment of dentin non-collagenous protein induction, indicating the significant potential of PDLSCs in the field of tooth root regeneration. Taking these backgrounds into consideration, results of our study explained why smokers had an unsatisfactory periodontal regeneration and provided target for clinical treatment in the near future.

α7 nAChR plays a critical role in regulating the process of immunoregulation and inflammatory reaction in our body [35] through which its specific agonist nicotine increases the expression of Ach to perform the anti-inflammatory function [36]. What is more, nicotine could also bind to α7 nAChR in immunological cells directly suppressing the expression of pro-inflammatory factors such as TNF-α and further regulate the NF-κB signaling pathway, thus influencing the process of inflammatory reaction [37]. It is concluded that α7 nAChR signaling pathway is an important anti-inflammatory pathway in many organs and systems.

However, previous results of our research group indicated that α7 nAChR also expressed in human periodontal ligament cells and nicotine could up regulate the expression of pro-inflammatory factor IL-1β through activating this receptor [9]. In animal experiments, Liu et al [38] found that nicotine deteriorated the alveolar bone loss of experimental periodontitis rats. Wang et al [9] further confirmed the mRNA and protein expression of α7 nAChR in periodontal tissues in rats, besides that the receptor could also be found in epithelial cells and osteoclast cells in walls of periodontal pockets of periodontitis rats. What was more, its expression was up regulated by stimulation of nicotine. All these results indicated that unlike its effect in other organs and systems, α7 nAChR might play a destructive role in the development of periodotntitis. Our study further confirmed that after binding to α7 nAChR, its specific agonist nicotine would affect the expression of osteogenic related genes and proteins of PDLSCs downstream, indicating that α7 nAChR expressed in PDLSCs took part in the down regulating procedure of nicotine toward the osteogenic potential of PDLSCs.

α-BTX, which is a specific antagonist of α7 nAChR, could reverse the impairment of osteogenic differentiation of PDLSCs after nicotine treatment, further confirming our conclusion that it is α7 nAChR through which nicotine deteriorates the osteogenic potential of PDLSCs. Based on the original assumption, with the stimulation of α-BTX, the protein expression of osteogenic makers should not be changed. However, results showed the tendency that when a-BTX was stimulated alone, the protein level of osteogenic makers decreased. We speculated that it is the toxicity of the chemical affected the protein expression of osteogenic makers during the long time procedure of osteogenic induction.

As described before, α7 nAChR played an important role in regulating inflammatory reactions [35] and in PDLCs, it is the up regulating expression of inflammatory factors through which the receptor mediating the destructive effect [9]. However, results of our research suggested that the activation of wnt signaling pathway after α7 nAChR was bound with its specific agonist nicotine; osteogenic differentiation of PDLSC was suppressed and the suppressive effects mediated by this pathway could be reversed after inhibiting wnt pathway by dkk1. Our results indicated that α7 nAChR might directly regulate wnt signaling pathway through regulating dkk1 and downstream GSK3β expression. However, the effect of inflammatory factors still need to be excluded in the future study.

Our research demonstrated that α7 nAChR mediated the down regulating effect of nicotine toward the osteogenic potential of PDLSC and protein expression of wnt related transcriptional factors could be detected in the downstream of α7 nAChR. With the stimulation of nicotine, wnt signaling pathway was activated and also took part in the negative regulation of nicotine toward the osteogenic potential of PDLSC. This result is coincident with the results of our previous studies which demonstrated that activating wnt pathway suppressed osteogenic differentiation of PDLSCs. However, more detailed mechanisms of wnt signaling pathway regulated by α7 nAChR should be further studied. What is more, whether non-canonical wnt pathway plays a certain role in the regulating process should also be confirmed.

## Supporting Information

Figure S1 **Differences of osteogenic differentiation capacity for hPDLSCs from 9 donor teeth.** A: hPDLSCs from 9 donor teeth were cultured in osteogenic medium and osteogenic differentiation was determined by Alizarin red staining after 21 days. Representative entire plate views of 6-well plates for PDLSCs from each donor; B: The quantity result for Alizarin Red S staining, there is no significant differences among PDLSCs from 9 donors.
(TIF)

Figure S2 **ALP staining of osteogenic differentiation of hPDLSC.** **A**: hPDLSCs treated by different dose of nicotien were cultured in osteogenic medium and osteogenic differentiation was determined by ALP staining after 7 days. Representative entire plate views of ALP staining in 6-well plates for PDLSCs from each group; **B**: hPDLSCs treated with/without nicotine and α-BTX were cultured in osteogenic medium and osteogenic differentiation was determined by ALP staining after 7 days. C: hPDLSCs treated with/without nicotine and dkk1 were cultured in osteogenic medium and osteogenic differentiation was determined by ALP staining after 7 days.
(TIF)

## References

[1] Armitage GC (2004) Periodontal diagnoses and classification of periodontal diseases. Periodontol 2000 34: 9–21. 14717852 10.1046/j.0906-6713.2002.003421.x

[2] Seo BM, Miura M, Gronthos S, Bartold PM, Batouli S, et al (2004) Investigation of multipotent postnatal stem cells from human periodontal ligament. Lancet 364: 149–155. 15246727 10.1016/S0140-6736(04)16627-0

[3] Ivanovski S, Gronthos S, Shi S, Bartold PM (2006) Stem cells in the periodontal ligament. Oral Dis 12: 358–363. 16792719 10.1111/j.1601-0825.2006.01253.x

[4] Calsina G, Ramon JM, Echeverria JJ (2002) Effects of smoking on periodontal tissues. J Clin Periodontol 29: 771–776. 12390575 10.1034/j.1600-051x.2002.290815.x

[5] Bergstrom J (2004) Tobacco smoking and chronic destructive periodontal disease. Odontology 92: 1–8. 15490298 10.1007/s10266-004-0043-4

[6] Kashiwagi Y, Yanagita M, Kojima Y, Shimabukuro Y, Murakami S (2012) Nicotine up-regulates IL-8 expression in human gingival epithelial cells following stimulation with IL-1β or P. gingivalis lipopolysaccharide via nicotinic acetylcholine receptor signalling. Arch Oral Biol 57: 483–490. 22119045 10.1016/j.archoralbio.2011.10.007

[7] Artis D (2008) Epithelial-cell recognition of commensal bacteria and maintenance of immune homeostasis in the gut. Nat Rev Immunol 8: 411–420. 18469830 10.1038/nri2316

[8] Tanaka H, Tanabe N, Suzuki N, Shoji M, Torigoe H, et al (2005) Nicotine affects mineralized nodule formation by the human osteosarcoma cell line Saos-2. Life Sci 77: 2273–2284. 15946696 10.1016/j.lfs.2005.02.022

[9] Wang XJ, Liu YF, Wang QY, Tsuruoka M, Ohta K, et al (2010) Functional expression of alpha 7 nicotinic acetylcholine receptors in human periodontal ligament fibroblasts and rat periodontal tissues. Cell Tissue Res 340: 347–355. 20309583 10.1007/s00441-010-0949-9

[10] Grando SA, Kawashima K, Wessler I (2003) Introduction: the non-neuronal cholinergic system in humans. Life Sci 72: 2009–2012. 12628450 10.1016/s0024-3205(03)00063-8

[11] Sakurai R, Cerny L, Torday J, Rehan V (2011) Mechanism for nicotine-induced up regulation of wnt signaling in human alveolar interstitial fibroblasts. Exp Lung Res 37: 144–154. 21133803 10.3109/01902148.2010.490288 PMC3062662

[12] Liu N, Shi S, Deng M, Tang L, Zhang G, et al (2011) High levels of b-Catenin signaling reduce osteogenic differentiation of stem cells in inflammatory microenvironments through inhibition of the noncanonical wnt pathway. Journal of Bone and Mineral Research 26: 2082–2095. 21638320 10.1002/jbmr.440

[13] Chen X, Hu C, Wang G, Li L, Kong X, et al (2013) Nuclear factor-κB modulates osteogenesis of periodontal ligament stem cells through competition with β-catenin signaling in inflammatory microenvironments. Cell Death and Disease 4: e510. 23449446 10.1038/cddis.2013.14 PMC3734811

[14] Kong X, Liu Y, Ye R, Zhu B, Zhu Y, et al (2013) GSK3β is a checkpoint for TNF-α-mediated impaired osteogenic differentiation of mesenchymal stem cells in inflammatory microenvironments. Biochim Biophys Acta 1830: 5119–5129. 23911749 10.1016/j.bbagen.2013.07.027

[15] Yang Z, Jin F, Zhang X, Ma D, Han C, et al (2009) Tissue engineering of cementum/periodontal-ligament complex using a novel three-dimensional pellet cultivation system for human periodontal ligament stem cells. Tissue Eng Part C Methods 15: 571–581. 19534606 10.1089/ten.tec.2008.0561

[16] Liu Y, Liu W, Hu C, Xue Z, Wang G, et al (2011) MiR-17 modulates osteogenic differentiation through a coherent feed-forward loop in mesenchymal stem cells isolated from periodontal ligaments of patients with periodontitis. Stem Cells 29: 1804–1816. 21898695 10.1002/stem.728

[17] Wang L, Shen H, Zheng W, Tang L, Yang Z, et al (2011) Characterization of stem cells from alveolar periodontal ligament. Tissue Eng Part A 17: 1015–1026. 21186958 10.1089/ten.tea.2010.0140

[18] Zhang J, An Y, Gao LN, Zhang YJ, Jin Y, et al (2012) The effect of aging on the pluripotential capacity and regenerative potential of human periodontal ligament stem cells. Biomaterials 33: 6974–6986. 22789721 10.1016/j.biomaterials.2012.06.032

[19] Chen SC, Marino V, Gronthos S, Bartold PM (2006) Location of putative stem cells in human periodontal ligament. J Periodontal Res 41: 547–553. 17076780 10.1111/j.1600-0765.2006.00904.x

[20] Gronthos S, Mrozik K, Shi S, Bartold PM (2006) Ovine periodontal ligament stem cells: isolation, characterization, and differentiation potential. Calcif Tissue Int 79: 310–317. 17033723 10.1007/s00223-006-0040-4

[21] Wada N, Menicanin D, Shi S, Bartold PM, Gronthos S (2009) Immunomodulatory properties of human periodontal ligament stem cells. J Cell Physiol 219: 667–676. 19160415 10.1002/jcp.21710

[22] Li B, Qu C, Chen C, Liu Y, Akiyama K, et al (2012) Basic fibroblast growth factor inhibits osteogenic differentiation of stem cells from human exfoliated deciduous teeth through ERK signaling. Oral Dis 18: 285–292. 22151351 10.1111/j.1601-0825.2011.01878.x PMC3292657

[23] Liu C, Li Y, Semenov M, Han C, Baeg GH, et al (2008) Control of beta-catenin phosphorylation/degradation by a dual kinase mechanism. Cell 108: 837–847. 10.1016/s0092-8674(02)00685-2 11955436

[24] World Health Organization (2005) Governments celebrate five years of anti-tobacco convention. Available: http://www.who.int/fctc/press.pdf. Accessed 2013 Jun 12.

[25] Luzzi LI, Greghi SL, Passanezi E, Santana AC, Lauris JR, et al (2007) Evaluation of clinical periodontal conditions in smokers and non-smokers. J Appl Oral Sci 15: 512–517. 19089190 10.1590/S1678-77572007000600011 PMC4327501

[26] Tomar SL, Asma S (2000) Smoking-atributable Periodontitis in the United States: findings from NHANES III. National Health and Nutrition Examination Survey. J Periodontol 71: 743–751. 10.1902/jop.2000.71.5.743 10872955

[27] Chambrone L, Chambrone D, Lima LA (2010) Predictors of tooth loss during long-term periodontal maintenance: a systematic review of observational studies. J Clin Periodontol 37: 675–684. 20528960 10.1111/j.1600-051X.2010.01587.x

[28] Wan CP, Leung WK, Wong MC, Wong RM, Wan P, et al (2009) Effects of smoking on healing response to non-surgical periodontal therapy: a multilevel modeling analysis. J Clin Periodontol 36: 229–239. 19236535 10.1111/j.1600-051X.2008.01371.x

[29] Iho S, Tanaka Y, Takauji R, Kobayashi C, Muramatsu I, et al (2003) Nicotine induces human neutrophils to produce IL-8 through the generation of peroxynitrite and subsequent activation of NF-kappaB. J Leukoc Biol 74: 942–951. 12960242 10.1189/jlb.1202626

[30] Makino A, Yamada S, Okuda K, Kato T (2008) Nicotine involved in periodontal disease through influence on cytokine levels. FEMS Immunol Med Microbiol 52: 282–286. 18205805 10.1111/j.1574-695X.2007.00373.x

[31] Liu Y, Zheng Y, Ding G, Fang D, Zhang C, et al (2008) Periodontal ligament stem cell mediated treatment for periodontitis in Miniature Swine. Stem Cells 26: 1065–1073. 18238856 10.1634/stemcells.2007-0734 PMC2653213

[32] Sonoyama W, Liu Y, Fang D, Yamaza T, Seo BM, et al (2006) Mesenchymal stem cell mediated functional tooth regeneration in Swine. PLoS One 20(1): e79. 10.1371/journal.pone.0000079 PMC1762318 17183711

[33] Zhang W, Abukawa H, Troulis MJ, Kaban LB, Vacanti JP, et al (2009) Tissue engineered hybrid tooth-bone constructs. Methods 47(2): 122–128. 18845257 10.1016/j.ymeth.2008.09.004

[34] Ma Z, Li S, Song Y, Tang L, Ma D, et al (2008) The biological effect of dentin noncollagenous proteins (DNCPs) on the human periodontal ligament stem cells (HPDLSCs) in vitro and in vivo. Tissue Eng Part A 14: 2059–2068. 18939934 10.1089/ten.tea.2008.0021

[35] Hamano R, Takahashi HK, Iwagaki H, Yoshino T, Nishibori M, et al (2006) Stimulation of alpha7 nicotinic acetyl-choline receptor inhibits CD14 and the toll-like receptor 4 expression in human monocytes. Shock 26: 358–364. 16980882 10.1097/01.shk.0000228168.86845.60

[36] Tracey KJ (2007) Physiology and immunology of the cholinergic anti-inflammatory pathway. J Clin Invest 117: 289–296. 17273548 10.1172/JCI30555 PMC1783813

[37] Yoshikawa H, Kurokawa M, Ozaki N, Nara K, Atou K, et al (2006) Nicotine inhibits the production of proinflammatory mediators in human monocytes by suppression of I-kappaB phosphorylation and nuclear factor-kappaB transcriptional activity through nicotinic acetylcholine receptor alpha 7. Clin Exp Immunol 146: 116–123. 16968406 10.1111/j.1365-2249.2006.03169.x PMC1809735

[38] Liu YF, Wu LA, Wang J, Wen LY, Wang XJ (2010) Micro-computerized tomography analysis of alveolar bone loss in ligature- and nicotine-induced experimental periodontitis in rats. J Periodontal Res 45: 714–719. 20572916 10.1111/j.1600-0765.2010.01290.x

