# Supplementary figures and images for: Nicotine Deteriorates the Osteogenic Differentiation of Periodontal Ligament Stem Cells through α7 Nicotinic Acetylcholine Receptor Regulating wnt Pathway
Source: PLoS One. 2013 Dec 20;8(12):e83102. doi: 10.1371/journal.pone.0083102 (PMC3869757; doi:10.1371/journal.pone.0083102)

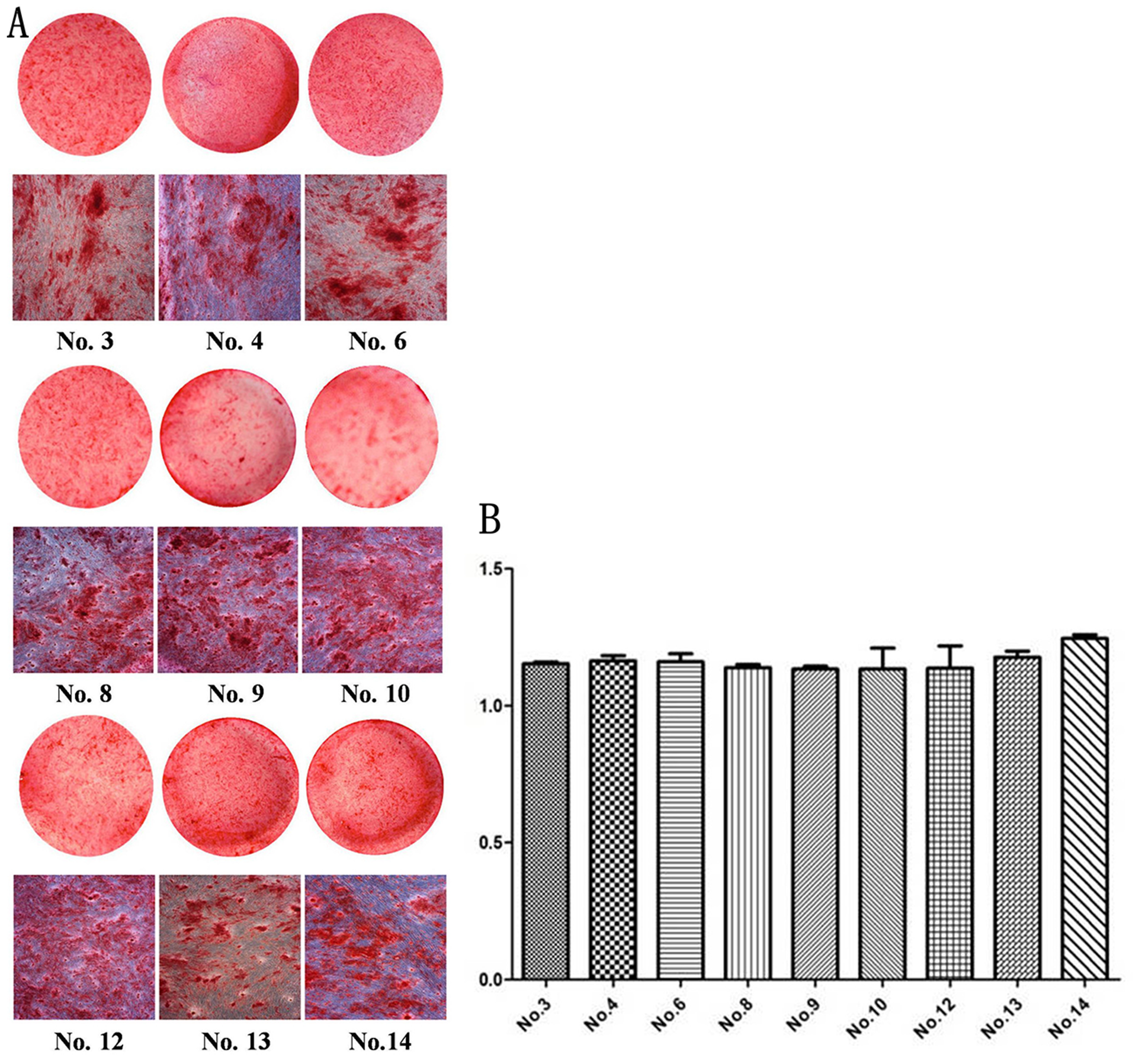

Supplement: Figure S1 — Differences of osteogenic differentiation capacity for hPDLSCs from 9 donor teeth. A: hPDLSCs from 9 donor teeth were cultured in osteogenic medium and osteogenic differentiation was determined by Alizarin red staining after 21 days. Representative entire plate views of 6-well plates for PDLSCs from each donor; B: The quantity result for Alizarin Red S staining, there is no significant differences among PDLSCs from 9 donors. (TIF) [file pone.0083102.s001.tif]

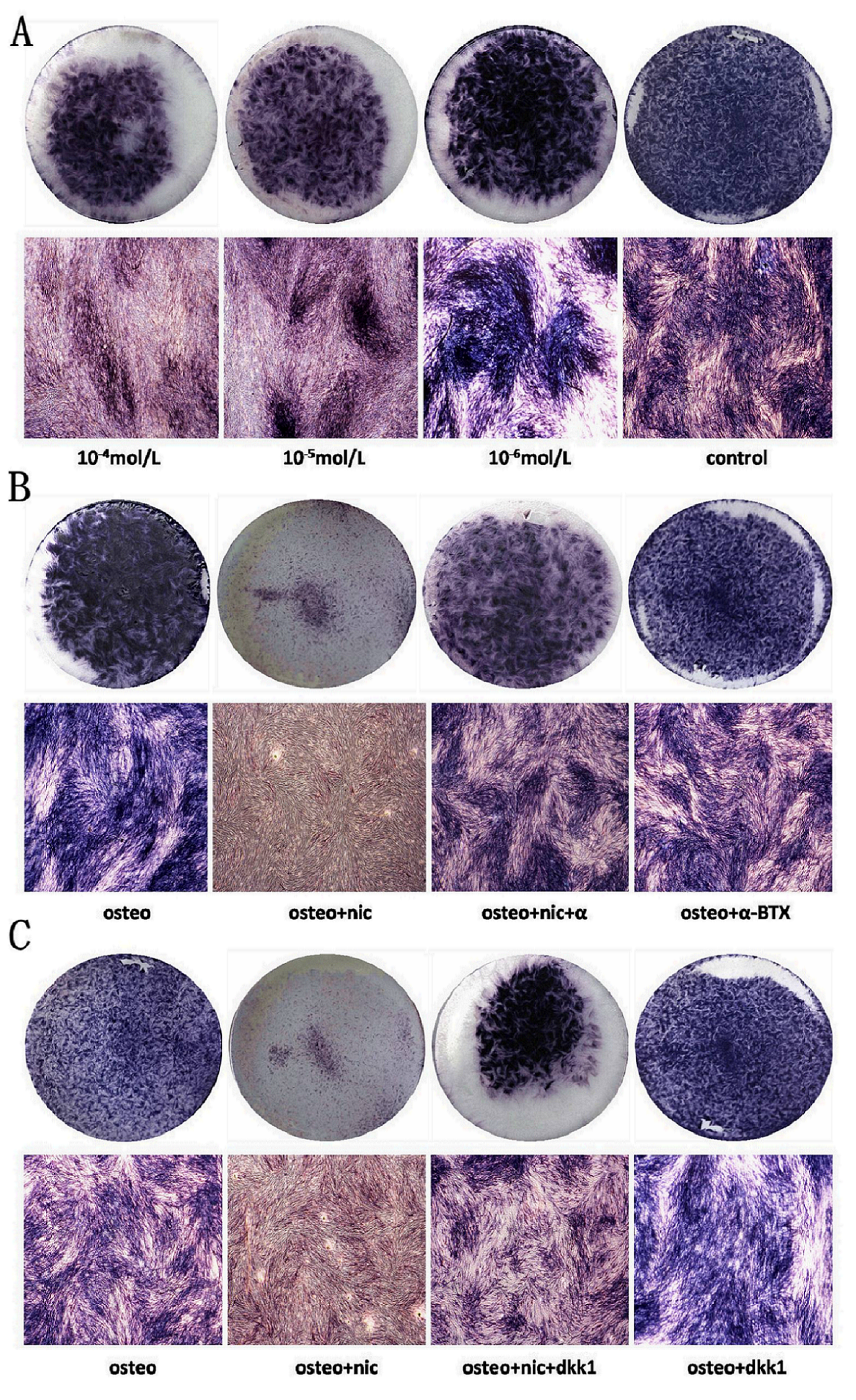

Supplement: Figure S2 — ALP staining of osteogenic differentiation of hPDLSC. A: hPDLSCs treated by different dose of nicotien were cultured in osteogenic medium and osteogenic differentiation was determined by ALP staining after 7 days. Representative entire plate views of ALP staining in 6-well plates for PDLSCs from each group; B: hPDLSCs treated with/without nicotine and α-BTX were cultured in osteogenic medium and osteogenic differentiation was determined by ALP staining after 7 days. C: hPDLSCs treated with/without nicotine and dkk1 were cultured in osteogenic medium and osteogenic differentiation was determined by ALP staining after 7 days. (TIF) [file pone.0083102.s002.tif]
